# The Penalties of Weakness

**Published:** 1888-03-10

**Authors:** 


### THE DOCTOR'S PULPIT.

### THE PENALTIES OF WEAKNESS.

SHOULD there be any penalties for weakness? Wickedness, all men agree, ought to be punished, but does not weakness come under a different category? One of the things all observant persons learn who live long enough is that there are many circumstances—some people call them laws—in the world which we do not quite clearly understand, and which we cannot always see the justice of. We know, however, that they do and will happen; and so, whether we think them just or not, we have to make up our minds to them. The penalties that attach to all kinds of weakness belong to this class. A man has a weak intellect, and is thereby rendered incapable of struggling successfully in the fight for existence. If his relatives cannot provide for him, the State must; and if the State does not, he will starve. The penalty of starvation attaches to his weakness of intellect. But he was born weak; could not help being so born; cannot by any effort he can make get rid of his disablement. Is it just, is it benevolent, that the poor helpless creature should starve? Starve, however, he must and will, unless, as we said, his relatives or the State feed him.

Another man comes into the world possessed with an evil spirit of selfishness. Everything he sees he desires to make his own. He cannot be made to understand that there is a great gulf fixed between *meum* and *tuum*, and that there are only two honest ways of crossing the gulf—purchase and gift. His want of fine moral preception leads him into a variety of difficulties which by no means agree either with his taste or convenience. Certain penalties result from his weakness, which he would much prefer to avoid; but they will not be avoided, as he sooner or later finds to his great disgust.

A woman of five-and-twenty discovers that her careful parents have by much self-denial left her with an ample provision against all possible worldly contingencies. She is under no necessity of working for daily bread; her servants are numerous enough to minister to every want; being young and fair, suitors are not wanting who are prepared to offer both heart and hand. But she is frivolous and wilful; has one mind to-day and another to-morrow; now prefers Mr. Smith and then Mr. Brown, and next day cannot endure either of them. She will be happy with a novel for an hour, and then miserable for the rest of the day, pleased neither with books, nor servants, nor Skye terriers, nor handsome lovers. In one thing only is she steadily consistent, and that is in a self-indulgence which knows no limits and no shame. Is she wicked? It would be honouring her too much to dignify her imbecile behaviour with the name of wickedness. She is merely weak. But wretched penalties flow from such weakness—penalties both physical, intellectual, and moral. She becomes languid and feeble in body, fitful and changeable in spirits; sometimes wild with frivolous excitement, and at other times plunged into contemptible despair. Her intellect gradually grows incapable of judgment, application, or any kind of original effort. Her morals are those of the spoilt and petted poodle, who snaps when he should look amiable, and looks amiable when he most deserves to be whipped and put into the street. Such a woman lives—a torment to herself, and a detestation to all who are unfortunate enough to be in any way associated with her. She dies without honour and without regret. What her hope may be in the future is best known to herself.

Of a very different class is the unhappy and much-to-be-pitied man who clearly sees the right and has no wish except to do it; but who from stress of circumstances is always spending too much on wife or family or friends. He knows that his income does not exceed five hundred a year, but to please his wife he pays a hundred for his house. He cannot afford to go to the theatre more than once a quarter, but Miss Spoylte, his second daughter, likes to go every week, and he cannot bear to deny her. His son is just eighteen, and ought to be at business; but Master Hightone hates business, and insists upon Oxford or the army. To Oxford he goes, and has not been there two terms before he has got deeply into debt. The father, who was nervous and irritable before, now loses his sleep; his strength and appetite fail; when he goes to business he dreads to see those whom he formerly met like an honest and straightforward Englishman. His health finally breaks down, and at the same time his creditors become too clamorous for endurance, and he has to do that which not one man in a thousand ever fully recovers from. He was not wicked; he was naturally a good and honourable man; but his fatal weakness has followed him with the penalties of shattered health, ruined fortune, and happiness destroyed.

These are familiar instances which might be illustrated from any reader's acquaintance. They are introduced, not because they are new, but because they help to give point and definition to the last examples to be quoted—examples which belong almost exclusively to the doctor's peculiar province. At Hastings, or any other winter resort, may be seen considerable numbers of persons of all ages being wheeled backwards and forwards along the parade in bath chairs. Some of these, both men and women, are old, gross, helpless, and almost fierce-looking, as if the strength, having deserted their legs and back, had all gone to intensify a temper never amiable, but now positively savage. All those aged people were once young, and many of them were strong, active, and lithe of limb—could dance or run, or ride across country with the best. Is it merely age which has reduced them to such a pitiable condition? In some it undoubtedly is, but in many it is nothing but weak self-indulgence: the unworthy habits of eating too much, drinking too much, and taking first of all too little exercise and then none at all. Their medical attendants have seen this state of things gradually approaching, but have feared to tell them what to expect. Perhaps in some cases an honest and faithful physician has spoken plainly, and has been dismissed for his pains. The writer knows of one medical man who was called in some years ago to a patient. He told his client that he took too much both to eat and drink, and did not exercise himself sufficiently; adding, that if he were to live upon sixpence a day, and earn it, for a couple of years, he would be a new man. That doctor was never called in again to that patient; but it is within the writer's knowledge that the patient is now almost at death's door, and is considerably under fifty years of age. He has no disease whatever, except what has resulted from his habits of eating, drinking, and idleness.

It is a thing of the first importance to learn in early life that weakness of body, of mind, of morals, of temper, and indeed of all kinds, is a disabling circumstance, and as surely leads to undesirable consequences as wickedness. Weakness in many cases is of such a kind that it may be countervailed by the exercise of other faculties of the mind or body. It is essential that a man understand his own physical and mental character, and, recognising his weaker parts, strive to develop them by exercise until they are stronger and more efficient. If brain, or stomach, or lungs, or liver, or legs, or memory, or will, or benevolence, or any other power be feeble, let the fact be admitted; let the weak part be strengthened by every possible means; let other faculties be called upon to supplement and help. Thus, and thus only, can a full, complete, and healthy life be lived; thus, and thus only, can old age be made pleasant and wholesome, and the end of life better than the beginning.

